# DNA Damage in Buccal Mucosa Cells of Pre-School Children Exposed to High Levels of Urban Air Pollutants

**DOI:** 10.1371/journal.pone.0096524

**Published:** 2014-05-02

**Authors:** Elisabetta Ceretti, Donatella Feretti, Gaia C V. Viola, Ilaria Zerbini, Rosa M. Limina, Claudia Zani, Michela Capelli, Rossella Lamera, Francesco Donato, Umberto Gelatti

**Affiliations:** 1 Unit of Hygiene, Epidemiology and Public Health, Department of Medical and Surgical Specialities, Radiological Sciences and Public Health, University of Brescia, Brescia, Italy; 2 Post-Graduate School of Public Health, University of Brescia, Brescia, Italy; Fondazione IRCCS Ca' Granda Ospedale Maggiore Policlinico, Università degli Studi di Milano, Italy

## Abstract

Air pollution has been recognized as a human carcinogen. Children living in urban areas are a high-risk group, because genetic damage occurring early in life is considered able to increase the risk of carcinogenesis in adulthood. This study aimed to investigate micronuclei (MN) frequency, as a biomarker of DNA damage, in exfoliated buccal cells of pre-school children living in a town with high levels of air pollution. A sample of healthy 3-6-year-old children living in Brescia, Northern Italy, was investigated. A sample of the children's buccal mucosa cells was collected during the winter months in 2012 and 2013. DNA damage was investigated using the MN test. Children's exposure to urban air pollution was evaluated by means of a questionnaire filled in by their parents that included items on various possible sources of indoor and outdoor pollution, and the concentration of fine particulate matter (PM10, PM2.5) and NO_2_ in the 1–3 weeks preceding biological sample collection. 181 children (mean age±SD: 4.3±0.9 years) were investigated. The mean±SD MN frequency was 0.29±0.13%. A weak, though statistically significant, association of MN with concentration of air pollutants (PM10, PM2.5 and NO_2_) was found, whereas no association was apparent between MN frequency and the indoor and outdoor exposure variables investigated via the questionnaire. This study showed a high MN frequency in children living in a town with heavy air pollution in winter, higher than usually found among children living in areas with low or medium-high levels of air pollution.

## Introduction

Air pollution is a global health problem, particularly in urban areas. Close to 90% of European citizens residing in urban areas are exposed to air pollution exceeding EU limit values (Air Quality Directive 2008/50/EC) and WHO guideline levels [1]–[3].

Several epidemiologic studies have demonstrated the association between air pollution exposure, especially to particulate matter, and mortality and morbidity in humans [4]–[8].

The finest fractions of particulate matter (PM2.5 and less) play a major role in causing chronic diseases because they are retained in the alveolar regions of the lungs and diffuse into the blood stream, inducing inflammation, oxidative stress, and blood coagulation [9], [10]. Extracts of urban air particles can induce cancer in animals [11], [12] and are mutagenic in bacteria, plant and mammalian cells in *in-vitro* tests [13]–[17]. *In-vivo* mutagenicity tests have been performed on humans as well. The micronuclei (MN) formation has been used as an indicator of chromosome damage, induced by substances that cause chromosome breakage (clastogens) as well as by agents that affect the spindle apparatus (aneugens) [18]. Other nuclear anomalies were investigated as biomarkers of DNA damage and cell death, which may be useful for a more comprehensive assessment of genotoxic damage [19]–[22].

An increase in cancer risk has been observed in the presence of a high level of chromosomal aberrations and micronuclei in several studies [23]–[26]. This biomarker can be investigated in various organs, tissues and body fluids, such as leukocytes or lymphocytes in peripheral blood, though cells derived from target tissues are considered more appropriate. In particular, exfoliated buccal and nasal cells have been used in the biological monitoring of people exposed to airborne pollutants as they are representative of epithelial respiratory tract cells and are easier to collect than those of other respiratory organs [21], [27]–[29].

An increase of micronuclei in leukocytes in peripheral blood has been observed in people exposed to urban air pollutants [30]–[36] and a strong correlation of MN frequency in buccal exfoliated cells and peripheral lymphocytes has been found [37], [38].

Children are a high-risk group in terms of the health effects of air pollution [2], [5], [39]–[42]. Some studies suggest that early exposure during childhood can play an important role in the development of chronic diseases in adulthood: the earlier the exposure, the greater the risk of chronic disease, including cancer [43].

Few studies have considered genetic damage in mucosa buccal cells as MN frequency in cells of children exposed to air pollution, and they only involved a small number of subjects showing cytogenetic damage in children or young adults living in polluted areas with a high concentration of PM or oxidant pollutants [33], [44]–[47].

The aim of this study was to investigate MN frequency, as a biomarker of DNA damage, in exfoliated buccal cells of pre-school children living in a town with high levels of air pollution during the winter season, when the highest levels of particulate matter and other pollutants are usually found.

## Materials and Methods

### Study design

This study is part of the RESPIRA study (Italian acronym for Rischio ESPosizione Inquinamento aRia Atmosferica), a molecular epidemiology cross-sectional study aiming to assess the presence of MN frequency in pre-school children living in Brescia, a highly polluted town in Northern Italy, located in the Po Valley, one of the most highly polluted areas of Europe. The children were recruited in 6 schools located in different areas of the town. The study enrolled children aged 3–6 years, born in Italy to European parents, without malignant tumours, who had not undergone radiotherapy or chemotherapy in the previous 12 months or X-rays in the previous 3 months.

The presence of MN and other nuclear anomalies were investigated in buccal mucosa cells taken from the children.

The biological samples were collected during two consecutive winter seasons (2012 and 2013) since the highest values of PM10 and PM2.5 in Brescia are usually observed in the winter months. The project was approved by the Ethics Committee of Local Unit Health of Brescia (Comitato Etico dell'ASL -Azienda Sanitaria Locale- di Brescia). The children's parents provided their written informed consent to participate in this study. All the data collected were treated confidentially in accordance with current Italian legislation (privacy law).

### Questionnaire

The children's parents were interviewed using an *ad hoc* questionnaire designed to gather information on exposure to air pollutants from both indoor and outdoor sources, including some characteristics of the area of residence (e.g. traffic, factories), parents' smoking habits, and children's respiratory diseases and drug consumption.

### Collection of air pollution data

Chemical data regarding daily concentration of the most commonly measured air pollutants (CO, NO_2_, SO_2_, benzene, O_3_, PM10 and PM2.5) were retrieved from the freely available ARPA (Regional Agency for Environmental Protection) database to characterize urban air quality.

### Collection of biological samples

All the biological samples were taken during or after a series of days with high levels of PM10, PM2.5 and NO_2_.

For the collection of buccal mucosa cells, the children rinsed their mouths twice with mineral water. Interdental brushes were used to collect epithelial buccal cells for the micronucleus test, by gently scraping the inside of both cheeks and dipping them into tubes containing 15 ml of PBS (phosphate buffered saline solution) [21]. This method is simple and non-invasive and therefore easily acceptable by both children and parents.

### Micronucleus test

After shaking and removing the brush, the epithelial buccal cells in PBS were centrifuged for 10 minutes at 1100 g at 4°C and re-suspended in warm PBS (37°C). In order to determine whether enough cells had been collected to perform the test, 10 µl of cell suspension was applied in a Burker chamber on which the number of cells was scored. The PBS cell suspension was then centrifuged for 4 minutes at 8700 g and the pellet was re-suspended in 700 µl of warm hypotonic solution (KCl 0.56%, 37°C). After 1 minute, 700 µl of cold methanol/acetic acid (14∶1, −20°C) was added. This fixed suspension was centrifuged for 4 minutes at 8700 g, and the pellet was re-suspended in 40 µl of warm PBS and dropped onto two frosted slides, which were dried and stained with Giemsa dye (5 minutes at room temperature). The slides were then washed with distilled water, dried and mounted with Eukitt.

The slides were examined at 1000X magnification for microscope analysis. Before MN frequency was assessed, cells were divided into two categories: “normal” cells and ones that are considered “abnormal” based on their cytobiological and nuclear features, which are indicative of DNA damage (MN and nuclear buds), cytokinetic failure (binucleated cells), proliferative potential (basal cells) or cell death (condensed chromatin, karyorrhexis, pyknotic, karyolitic and without nucleus cells), according to the Buccal Micronucleus Cytome (BMCyt) assay [22]. To assess MN, nuclear buds, binucleated and basal cells frequency, at least 2000 “normal” cells per slide (two slides per subject, 4000 cells per subject) were scored by two expert operators with duplicate reading. The results are given as the percentage of cells with MN and buds, and binucleated and basal cells [19], [22]. Moreover, condensed chromatin, karyorrhexis, pyknotic, karyolitic, and without nucleus cells were evaluated scoring 2000 total cells per subject, and expressed as percentages.

### Statistical analysis

All the data were processed to investigate the associations between air pollution parameters and MN frequency. As buccal cells have a short life, no more than 3 weeks [22], the associations between frequency of MN and other biomarkers frequency and air pollutants concentration (CO, NO_2_, SO_2_, benzene, O_3_, PM10 and PM2.5) were analysed using air pollutants mean levels at 0 (sampling day), 1, 2 and 3 weeks before sampling. Both univariate analysis and multivariate analysis (multiple regression, logistic regression) were performed to assess the association investigated, adjusting for confounding factors. Particularly, linear regression with biological parameters as dependent variables and the concentration of air pollutants as independent variables were fitted. In order to improve the interpretation of these findings the coefficients of the linear regression were computed on a 10 µg/m^3^ scale of measures of air pollutants. Two-tailed statistical tests were performed, with 0.05 p-value as the threshold for rejecting the null hypothesis. All the analyses were performed using the Stata TM 12.0 statistical package (Stata Statistical Software Release 12.0, 2012; Stata Corporation, College Station, Texas, USA).

## Results

A total of 222 children were recruited, of whom 181 (mean age ±SD: 4.35±0.84 years; 56.9% males) were examined because 41 samples were not eligible for MN analysis due to an insufficient number of cells collected.

The mean±SD MN frequency expressed as a percentage was 0.29±0.13 (median 0.28 and range 0.085–0.990).

Table 1 shows the results of the MN analysis, according to children's socio-demographic characteristics and habits, and indoor and outdoor exposure data. No variable was associated with MN frequency in exfoliated buccal cells.

**Table 1:** Micronuclei (MN) frequencies observed in buccal mucosa cells of children according to socio-demographic features and exposure variables (N = 181).

Subjects and demographic features	N (%)	% MN (Mean±SD)
Sex		
M	103 (56.9)	0.30±0.13
F	78 (43.1)	0.29±0.13
Children's age		
3 years	34 (18.8)	0.31±0.16
4 years	59 (32.6)	0.28±0.13
5–6 years	88 (48.6)	0.29±0.12
Parents' education (at least one parent)		
Primary school or less	19 (10.5)	0.28±0.12
Secondary school	53 (29.3)	0.32±0.16
College or university	109 (60.2)	0.28±0.12
Home characteristics		
Traffic in the area		
Heavy	97 (53.6)	0.31±0.14
Moderate	59 (32.6)	0.28±0.13
Very light	25 (13.8)	0.25±0.08
Truck traffic in the area		
Heavy	31 (17.1)	0.30±0.12
Moderate	67 (37.0)	0.31±0.17
Very light	82 (45.3)	0.27±0.09
Indoor exposure		
Gas stove in home	61 (33.7)	0.28±0.11
Fireplace in home	37 (20.4)	0.28±0.11
Presence of smokers in home	28 (15.5)	0.30±0.18
School characteristics		
Traffic in the area		
Heavy	102 (56.4)	0.31±0.15
Moderate	68 (37.6)	0.27±0.09
Very light	10 (5.5)	0.29±0.15
Truck traffic in the area		
Heavy	27 (14.9)	0.33±0.19
Moderate	80 (44.2)	0.29±0.12
Very light	72 (39.8)	0.28±0.12
Child's habits		
Plays outdoors		
Less than 1 hour	78 (43.1)	0.31±0.16
More than 1 hour but less than 3 hours	67 (37.0)	0.28±0.10
3 hours or more	35 (19.3)	0.28±0.10
Remains in the kitchen while meals are cooked		
Never	24 (13.3)	0.29±0.13
Sometimes	119 (65.8)	0.30±0.14
Often/always	37 (20.4)	0.27±0.09
Consumes fried/grilled/smoked food		
Never	5 (2.8)	0.36±0.17
More than once per month	123 (68.0)	0.28±0.14
Parents' smoking habits		
Neither parent smoke	118 (65.2)	0.29±0.12
Mother smoked during pregnancy	40 (22.1)	0.29±0.14
Mother smokes	30 (16.6)	0.29±0.15
Father smoke	49 (27.1)	0.30±0.17
Both parents smokers	42 (23.2)	0.29±0.15

Table 2 sets out all the biomarkers evaluated in the buccal mucosa cells of children according to children's sex and age. No statistically significant difference was observed according to sex and age for each parameter.

**Table 2:** Frequency of micronuclei (MN) and other nuclear anomalies in all children and according to sex and age.

	N° children	% MN	% Nuclear buds	% Binucleated cells	% Basal cells	% Condensed chromatin cells	% Karyorrhetic cells	% Pyknotic cells	% Karyolytic cells	% Without nucleus cells
		(Mean±SD)	(Mean±SD)	(Mean±SD)	(Mean±SD)	(Mean±SD)	(Mean±SD)	(Mean±SD)	(Mean±SD)	(Mean±SD)
All children	181	0.29±0.13	0.02±0.04	0.11±0.08	0.72±1.15	17.20±6.13	7.44±4.29	0.57±0.48	3.72±3.37	1.42±1.28
Sex										
Males	103	0.30±0.13	0.02±0.04	0.11±0.09	0.84±1.38	17.10±6.26	7.88±4.72	0.61±0.50	3.96±3.97	1.42±1.18
Females	78	0.29±0.13	0.02±0.03	0.10±0.08	0.56±0.73	17.33±6.00	6.86±3.60	0.52±0.46	3.42±2.34	1.43±1.40
Children's age										
3 years	34	0.31±0.16	0.02±0.06	0.10±0.09	0.56±0.80	16.70±4.52	7.48±4.84	0.65±0.53	4.07±2.91	1.65±1.27
4 years	59	0.28±0.13	0.01±0.02	0.13±0.10	0.64±0.69	16.58±6.18	7.96±4.41	0.52±0.49	3.21±2.31	1.23±1.15
5–6 years	88	0.29±0.12	0.02±0.03	0.09±0.06	0.83±1.46	17.80±6.62	7.07±3.99	0.57±0.46	3.94±4.05	1.47±1.35

**Figure 1:**
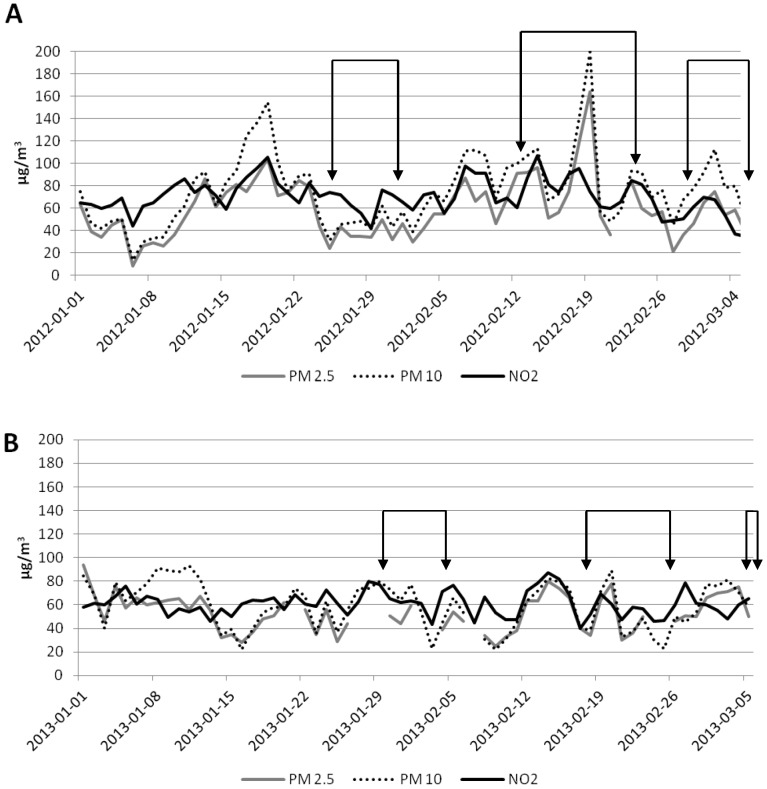
PM10, PM2.5 and NO_2_ concentration in 2012 (A) and 2013 (B). The arrows indicate the days on which biological sampling was performed. The dotted lines refer to the days without measurement of air pollutants.

The daily levels of PM10, PM2.5 and NO_2_ from January to March 2012 and 2013, and biological sampling days are shown in Figure 1. During these months, the concentrations of PM10 and PM2.5 were almost always over the EU limit values for daily means (50 and 25 µg/m^3^, respectively). Likewise, the annual EU limit for NO_2_ (40µg/m3) was exceeded on all the days. On the contrary, the concentration of CO, ozone, SO_2_ and benzene remained low throughout the period considered and were always below the EU limit values (data not shown).

The mean MN frequency according to concentrations of PM10, PM2.5 and NO_2_ on the day of sampling and one, two and three weeks before sampling are shown in Table 3. A fair variation (from 0.21% to 0.62%) was found in MN frequency from one day to another, without a clear relationship with concentration of air pollutants.

**Table 3:** Micronuclei (MN) frequency and PM10, PM2.5 and NO_2_ concentrations on the day of sampling and in the 1–3 weeks preceding biological sampling.

			Same day			Weeks preceding biological sampling								
Day of		% MN				1 week			2 weeks			3 weeks		
sampling	N° children	(Mean±SD)	PM10	PM2.5	NO_2_	PM10	PM2.5	NO_2_	PM10	PM2.5	NO_2_	PM10	PM2.5	NO_2_
			(µg/m^3^)	(µg/m^3^)	(µg/m^3^)	(µg/m^3^)	(µg/m^3^)	(µg/m^3^)	(µg/m^3^)	(µg/m^3^)	(µg/m^3^)	(µg/m^3^)	(µg/m^3^)	(µg/m^3^)
2012-01-24	8	0.62±0.23	52.0	44	70.3	110.2	82.6	84.5	92.9	73.8	80.0	73.9	59.6	74.1
2012-01-25	4	0.27±0.10	31	24	73.9	99.8	78.1	82.0	93.0	74.4	79.3	74.4	60.0	74.6
2012-01-26	4	0.30±0.14	45.5	43	71.8	84.7	68.7	78.9	90.8	72.4	78.4	73.5	59.0	75.1
2012-01-30	4	0.18±0.03	62.0	50	76.1	50.9	42.0	65.4	80.7	62.4	74.6	76.2	60.7	74.7
2012-01-31	7	0.27±0.10	43.0	32	71.9	46.8	37.9	64.5	78.5	60.2	74.5	77.5	61.8	74.9
2012-02-13	12	0.26±0.15	106.5	92	87.2	97.1	72.0	77.4	77.1	58.1	72.5	68.4	52.8	70.2
2012-02-14	12	0.34±0.08	113.0	96	107.1	100.1	75.0	80.1	80.3	61.1	73.3	69.1	53.4	70.4
2012-02-20	12	0.25±0.09	57.0	53	61.3	112.3	93.1	87.0	104.7	82.6	82.2	88.9	69.8	77.4
2012-02-22	11	0.29±0.04	57.5	nd	65.4	95.9	79.0	76.6	98.2	67.3	79.0	88.9	70.1	76.1
2012-02-23	10	0.23±0.05	93.5	83	84.4	94.6	71.7	74.4	94.3	72.9	77.2	88.9	68.0	76.1
2012-02-28	11	0.24±0.09	67.5	36	50.5	68.7	44.3	65.0	87.0	65.9	74.2	91.4	69.0	76.1
2012-02-29	12	0.30±0.13	77.5	46	60.8	71.5	44.3	63.7	83.7	61.6	70.1	89.3	66.5	73.9
2012-03-01	12	0.24±0.07	92.0	65	69.7	74.4	50.9	63.3	84.5	61.3	68.7	87.7	65.6	72.5
2012-03-05	6	0.25±0.04	51.0	38	34.5	78.8	50.7	55.6	74.6	49.8	61.2	87.2	64.2	69.8
2013-01-29	6	0.40±0.19	80	nd	77.4	57.9	36.7	63.8	53.6	40.1	62.5	61.3	46.4	60.0
2013-01-30	3	0.21±0.03	73.5	51	65.5	59.8	32.8	66.2	56.5	40.5	64.5	60.8	45.6	60.6
2013-02-04	1	0.32±0.00	45.0	39	71.4	63.7	38.5	64.7	60.8	41.6	63.4	55.0	41.7	62.1
2013-02-18	9	0.31±0.05	39.0	34	52.2	65.2	60.6	67.9	50.3	46.8	64.3	54.8	44.9	64.4
2013-02-19	5	0.33±0.06	71.0	64	69.5	64.4	60.0	68.6	49.9	46.4	62.9	53.1	46.8	63.1
2013-02-25	7	0.41±0.15	23	nd	46.9	58.5	48.7	55.8	55.7	55.1	61.8	50.1	47.8	61.4
2013-03-04	16	0.25±0.07	70.5	75	60.1	58.0	59.0	58.6	53.9	53.8	57.2	57.7	56.3	60.7
2013-03-05	9	0.21±0.04	58.0	50	65.0	64.8	61.3	60.5	56.1	57.3	57.8	58.9	58.3	61.4

The coefficients of linear regressions of the biological markers on the concentration of PM10, PM2.5 and NO_2_ on the same day and at 1, 2 and 3 weeks preceding biological sampling computed for 10 µg/m^3^ units of increase are shown in Table 4. A modest, though statistically significant, increase of the frequency of nuclear anomalies (MN, buds and binucleated cells) and of basal cells for an increase of PM10, PM2.5 and NO_2_ was found. No clear pattern was evident for the other parameters.

**Table 4:** Coefficients of linear regression of the biological markers for 10 µg/m^3^ units of increase of PM10, PM2.5 and NO_2_ concentration.

	Same day			Weeks preceding biological sampling								
				1 week			2 weeks			3 weeks		
	PM 10	PM 2.5	NO_2_	PM 10	PM 2.5	NO_2_	PM 10	PM 2.5	NO_2_	PM 10	PM 2.5	NO_2_
MN	−0.01	−0.00	0.00	0.01*	0.01	0.03**	0.00	0.00	0.02	−0.01	−0.03**	−0.01
Nuclear buds	−0.00	0.00	0.08*	0.01***	0.01**	0.01***	0.00	0.00	0.01**	−0.00	−0.00	0.00
Binucleated cells	−0.00	−0.00	0.01	0.01**	0.01*	0.02**	0.01**	0.02**	0.03***	0.00	0.00	0.03**
Basal cells	0.02	0.00	0.10	0.20***	0.15**	0.40***	0.15**	0.26**	0.42***	0.06	0.03	0.40**
Condensed chromatin cells	0.20	0.06	1.07***	1.13***	1.06***	2.93***	1.26***	1.94***	3.61***	0.74*	0.71	3.79***
Karyorrhetic cells	0.04	−0.36**	−0.26	1.73	−0.29	0.20	0.66***	0.60	1.22**	1.05***	1.32**	2.22***
Pyknotic cells	0.01	0.04**	0.02	0.02	0.03	0.03	−0.03	−0.01	−0.05	−0.07**	−0.11**	−0.12*
Karyolytic cells	−0.04	0.02	−0.25	−0.48***	−0.36**	−0.93***	−0.61***	−0.93***	−1.40***	−0.61**	−0.71*	−2.05***
Without nucleus cells	0.09*	0.07	0.12	−0.01	−0.08	0.05	−0.01	−0.04	0.03	−0.01	−0.12	−0.04

statistically significant: *p<0.05;

**p<0.01;

***p<0.001.

## Discussion

The main finding of this study was a surprisingly high level of MN frequency in exfoliated buccal cells of pre-school children living in Brescia.

The MN frequency (mean: 0.29%) observed in our study was higher than that observed in healthy children “without important exposure” (0.108%) as a result of a pooled analysis of 321 children aged up to 9 years [48]. Furthermore, the value observed in children living in Brescia was higher than that shown in adolescents or young adults working in an engine repair workshop (mean age: 15.5 years, MN frequency: 0.07%) [49] or exposed to ozone air pollution (university students, MN frequency: 0.12%) [46]. However, these studies are not comparable to ours due to the different age of the people investigated. Age is in fact one of the most important factors affecting MN data in both lymphocytes and oral cells, with a progressively increase of MN with age [33], [36], [48], [50]. However, the MN frequency observed in our study was higher than that found in children (aged 6–17) living in the urban area of Calcutta [44], which was higher than that found in those living in rural area (0.22% vs 0.17%). Children of a similar age to those in our study were included in a study on genotoxicity of air pollutants generated by biomass burning [47], which found a significant difference in MN frequency in oral cells between children exposed to high PM2.5 concentration and those living in a control area with a lower PM2.5 concentration (0.12% vs 0.02% in children under 7 years of age). A MN frequency similar to, or higher than, that observed in our study was reported in some studies carried out in Eastern Europe, as revised by Holland et al. [36].

According to a wide dataset which includes adults and children, a MN frequency interval of between 0.03% and 0.17% can be considered as a range of spontaneous MN frequency in buccal exfoliated cells of “unexposed” children [48]. Other authors have reported a range of 0.05–0.08% as the baseline MN frequency in exfoliated cells in healthy people [46]. Therefore, the mean MN frequency of 0.29% found in our study is about two-three-fold higher than that considered as a “reference” value for children of this age.

The MN frequency did not vary according to sex, age, parental education and all the variables investigated through the questionnaire. A weak, though statistically significant, association of MN frequency with concentration of air pollutants in the week preceding the buccal mucosa cell collection, but not on the same day and in the second or third week before sampling, was found. These results are partially in agreement with findings from previous studies comparing people at different exposure levels to air pollutants, which showed that industrial or urban air pollutants had a genotoxic effect on mucosa cells [44], [47], [49]. It should be pointed out, however, that the concentration of PM10, PM2.5 and NO_2_ was high throughout the study period, and always above the EU proposed limits for daily levels. The range of variation of pollutant concentration may therefore have been too narrow to determine a substantial change in MN frequency in the children. Alternatively, a threshold instead of a dose-response mechanism may be proposed, so similar genotoxic effects on children's buccal mucosa cells may have been produced by air pollutant values above a defined level. Lastly, the role of confounding factors, such as children's diet, physical activity and others, which was not investigated in our study, cannot be excluded. Indeed, some studies have observed an association between MN frequency in buccal mucosa cells and diet (e.g. fruit consumption, supplementation with B vitamins or antioxidants) and some specific behaviours, such as smoking habit, intake of alcohol, and betel quid chewing [21], [37], [48], [51], [52].

Other nuclear anomalies apart from MN were associated with the PM10, PM2.5 and NO_2_ concentration, particularly during the week preceding exfoliated cells sampling confirming findings of MN analysis on the possible effects of air pollutant exposure. However, the biological significance of these biomarkers in exfoliated oral cells is still unclear. Some nuclear anomalies, as buds, could be the result of genotoxic damage but these events are also associated with natural degenerative processes in these short-lived cells [53], [54]. The frequency of binucleated cells was related to air pollutants concentration too. This parameter is primarily an indicator of cytotoxicity because of failures in cell division. Although these findings should be considered with caution because of paucity of data on the relationship between these biomarkers and air pollutants levels, overall they suggest that air pollution exposure may induce both genotoxic and cytotoxic damage in buccal mucosa cells of children. This observation strengthens the need to investigate these additional biomarkers of DNA damage together with MN for a more comprehensive evaluation of these issues.

This study has various strengths. First, the number of subjects recruited (181 children) is relatively high compared to previous studies of MN frequency in oral cells, particularly in this age group.

Second, it is unlikely that other important factors biased our results: there is no major industrial exposure in the area and few children could have been exposed to indoor pollution sources, including passive smoking at home, according to data collected via the questionnaire. Furthermore, the inclusion and exclusion criteria, e.g. residence in town, born to European parents, no malignant tumours, no radiotherapy or chemotherapy in the previous 12 months, no X-ray exposure in the three months before buccal cell collection, allowed us to rule out a possible role of other mutagenic factors.

Third, the collection of exfoliated buccal cells during two consecutive winter seasons (2012 and 2013) provided information on two periods characterized by similar environmental conditions and it showed consistent results, with no substantial difference in MN frequency in the two years.

Lastly, 4000 cells per child were scored for assessing MN frequency, representing a very high number of observations, higher than normally performed in current practice, in order to reduce the variability of the estimate, as suggested by Ceppi et al. [37].

This study has some limitations, however, mainly the lack of a control group of subjects living in a less polluted area or another collection of biological samples from the same or similar children in a period with lower airborne pollutant levels, for evaluating the role of spatial or seasonal differences in influencing MN frequency in buccal mucosa cells. Differences between more and less polluted areas have been found in some research [47] and seasonal differences in air pollution composition could be relevant for genotoxic damage, such as the summer increase of ozone concentration [46]. Nevertheless, this study was designed to assess MN frequency in a sufficiently large sample of children regularly exposed to high levels of air genotoxic pollutants (PM and PAH), and not seasonal differences. We are, however, planning to extend the research to summer months and to children living in less polluted areas.

Only one sampling of oral cells was performed for each child, so the intra-individual variability of this early effect biomarker was not evaluated. The use of only one effect measure does not cause an important bias, however, because it reflects the mean exposure of the three preceding weeks, when the climate situation had not substantially changed. On the other hand, previous studies which found an association between this biomarker and air pollution also used a single measure of effect [31], [44], [47], [49], [50], [55], [56].

MN are a marker of early biological effect able to detect both clastogens and aneuploidy-inducing chemicals [18]. They are formed from acentric chromosomal fragments or whole chromosomes that are not included in the main daughter nuclei during nuclear division and their induction therefore reflects clastogenic and/or aneugenic events. They represent stable cytogenetic alterations which are the result of recent exposure of buccal mucosa cells, in a rapidly dividing tissue [18], [28]. These cells are short-lived and they are the first barrier for substances introduced into the body by inhalation or ingestion and may be an excellent target tissue for detecting early genotoxic effects induced by mutagenic airborne compounds. Their use can, therefore, be proposed for assessing exposure to airborne mutagens, especially in the paediatric population as they are easy to collect, considering also the strong correlation of MN frequency in these cells and in lymphocytes, which have been shown to be related to the subsequent risk of developing cancer [37], [48].

Brescia is a highly industrialized area with a high level of motor vehicle traffic. It is located in the Po Valley, one of the most highly polluted areas of Europe, where the concentrations of PM10, PM2.5 and NOx are usually above the EU reference values for many days of the year, as in the two years of this study (2012 and 2013), similar to those found in other towns and cities in the Po Valley [3].

In conclusion, this study shows that children living in a town with high levels of air pollutants in a Western country have a high level of MN in buccal mucosa cells, confirming previous findings of a mutagenic effect of urban air pollution on human beings.
